# Polyphenol Intake from Herbs and Spices

**DOI:** 10.3390/nu17152445

**Published:** 2025-07-27

**Authors:** Cynthia Blanton

**Affiliations:** Department of Nutrition and Dietetics, Idaho State University, Pocatello, ID 83201-8117, USA; cynthiablanton@isu.edu; Tel.: +1-(208)-282-3937

**Keywords:** herbs, spices, (poly)phenols, questionnaire

## Abstract

**Background**: Culinary herbs and spices are potent sources of bioactive compounds such as (poly)phenols that confer health benefits to consumers. Observational studies have quantified (poly)phenol intake levels from foods and beverages but not herbs and spices. Hence, the contribution of herbs and spices to (poly)phenol intakes is unclear. **Methods**: The current study measured herb and spice total (poly)phenol consumption in a convenience sample of adults (*n* = 212) using a validated online herb and spice questionnaire. Respondents reported the frequency and amount of consumption of 27 herbs and spices during the past month. Total (poly)phenol concentration (mg) for each herb and spice was calculated using the online database Phenol-Explorer. **Results**: Responses showed monthly intakes of 679.92 (1134.06) (median, IQR) mg total (poly)phenols from 47.44 (60.71) g herbs and spices. Cinnamon, black pepper and cloves were the largest contributors to total (poly)phenol intakes from herbs and spices. **Conclusions**: These findings suggest that herbs and spices contribute potentially meaningful amounts of (poly)phenols to total dietary (poly)phenol intakes and that existing reports of (poly)phenol consumption for populations may underestimate actual levels by 3–12%.

## 1. Introduction

Culinary herbs and spices have remained important contributors to traditional diets and across cultures throughout history. The health properties of herbs and spices have been recognized for centuries and are the focus of current research [1,2,3]. (Poly)phenols and other compounds in herbs and spices exhibit antioxidant, anti-inflammatory activities that are associated with improvements in glucose and lipid metabolism, cardiovascular health, and neurodegenerative conditions [4]. Multiple large-cohort studies demonstrate significant beneficial effects of dietary (poly)phenols on health outcomes and mortality risk [5]. For example, in the European Prospective Investigation into Cancer and Nutrition-InterAct case–cohort study with 3.99 million person-years of follow-up, higher versus lower intakes of flavonoids, a class of (poly)phenols, were associated with a significantly reduced risk of type II diabetes [6]. Examination of the PREDIMED cohort including 7172 individuals showed ~50%-lower risks of cardiovascular disease with higher intakes of (poly)phenols and specific (poly)phenol subclasses [7]. Lower risks of metabolic syndrome and its components were shown with higher intakes of (poly)phenols in the Polish arm of the Health, Alcohol and Psychosocial factors In Eastern Europe (HAPIEE) cohort of 8821 participants [8]. However, the dietary assessment methods used in these studies do not query culinary herb and spice use.

Considering the high concentration of (poly)phenols in herbs and spices and their widespread use during food preparation and at meals, the lack of data on (poly)phenol intakes from herbs and spices raises concerns about the completeness of (poly)phenol assessment [9,10]. Incomplete measurement of (poly)phenol intakes can introduce error into research that seeks to determine relationships between (poly)phenol consumption and health outcomes. The current study sought to provide empirical data on the level of total (poly)phenols consumed in herbs and spices. The results are intended to inform decisions about including herbs and spices in dietary assessments of (poly)phenol intake.

## 2. Materials and Methods

The study was approved by the Idaho State University Institutional Review Board under protocol identifier IRB-FY2024-222. Men and women of age ≥ 18 years were eligible to participate. A convenience sample of participants was recruited using online university announcements and social media between August and October 2024. The incentive for completing the questionnaire was entry into a draw for one of several gift cards. Individuals provided consent to participate in the study by completing an online consent form that preceded access to the herb and spice questionnaire. The text of this article was composed using Microsoft® Office Word version 2108.

### 2.1. Herb and Spice Questionnaire

The herb and spice questionnaire has been validated for content and comparison against a criterion method [11]. The original questionnaire was modified for the current study to include only those herbs and spices for which (poly)phenol content was available from the online database Phenol-Explorer version 3.6 (*n* = 27 herbs and spices; Table 1) [12].

Questionnaire options for frequency of use were as follows:

- Never;
- Less than once per month;
- 1, 2, or 3 times per month;
- 1, 2, 3, 4, 5, or 6 times per week;
- 1, 2, or >3 times per day.

Options for portion size were as follows:

- <1/2 teaspoon, 1, 2, or >3 teaspoons;
- 1, 2, 3, 4, or >5 shakes, with 3 options for shaker hole size (small, medium and large). Hole sizes were depicted in photographs.
- 1, 2, 3, 4, or >5 turns of a grinder. Photo of pepper grinder was shown.
- “Not sure because it is added during food preparation” and “Not sure/I don’t pay attention” were included in frequency and portion size questions to reduce the likelihood of guessing.

### 2.2. (Poly)phenol Calculations

Information gathered from the herb and spice questionnaire included total number of herbs and spices consumed and frequency and portion size used per month. For each participant, reported herb and spice intake was converted to grams (g) consumed per month (mo) using measurements collected by a calibrated digital scale. These measurements were obtained for each herb and spice for each portion size, e.g., number of shakes per shaker hole size or teaspoon. For “Not sure” responses, a portion size of 0.5 tsp was assumed. Herbs and spices used for measurements were purchased from local grocery stores. Total (poly)phenol amount in mg per 100 g herb/spice weight as measured using the Folin assay was obtained from Phenol-Explorer version 3.6, and from this value, the total (poly)phenol amount for each herb and spice consumed per month per participant was calculated [12]. Total (poly)phenol amount consumed per month from all herbs and spices per participant was also calculated. For ease of comparison with other published data, the following were calculated by dividing each respondent’s values by 30, the average number of days in a month: herb/spice consumed in g/d, total (poly)phenols from individual herbs/spices in mg/d, and total (poly)phenols consumed from all herbs/spices in mg/d. Polyphenol subclass intakes were obtained from Phenol-Explorer and expressed as mg consumed per month.

### 2.3. Data Analysis

Data distributions were analyzed using histograms and the Shapiro–Wilk test and intakes of herbs/spices and (poly)phenols were non-normally distributed. Descriptive data are therefore presented as medians with interquartile ranges. Means with standard deviations are also presented for ease of comparison with findings from other published studies. The Kruskal–Wallis test, followed by nonparametric comparisons for each paired Wilcoxon method, was used to compare the (poly)phenol intakes from specific herbs and spices. A *p* value of <0.05 was considered statistically significant. Analyses were performed using JMP^®^, Version 18, SAS Institute Inc., Cary, NC, USA, 1989–2025.

## 3. Results

From 233 total questionnaire attempts, 212 were complete and used in analysis. Total intakes of herbs and spices per month and day are shown in Table 2. The consumption rates of each herb and spice are presented in Table 3. Black pepper, garlic, and cinnamon were the most commonly consumed, each by more than 64% of respondents. Black pepper and garlic were consumed in the greatest amounts (Table 4). The number of herbs and spices consumed monthly by respondents ranged from 1 to 21, with the median (IQR) being 8.00 (8.00), mean (SD) 8.34 (5.14), and mode 10.

**Figure 1:**
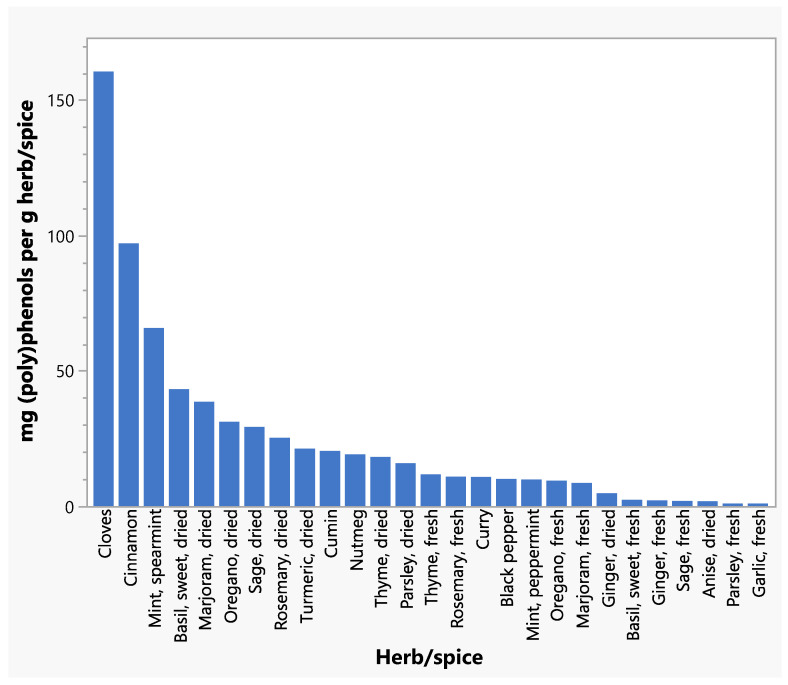
Total (poly)phenol concentrations for individual herbs and spices.

**Figure 2:**
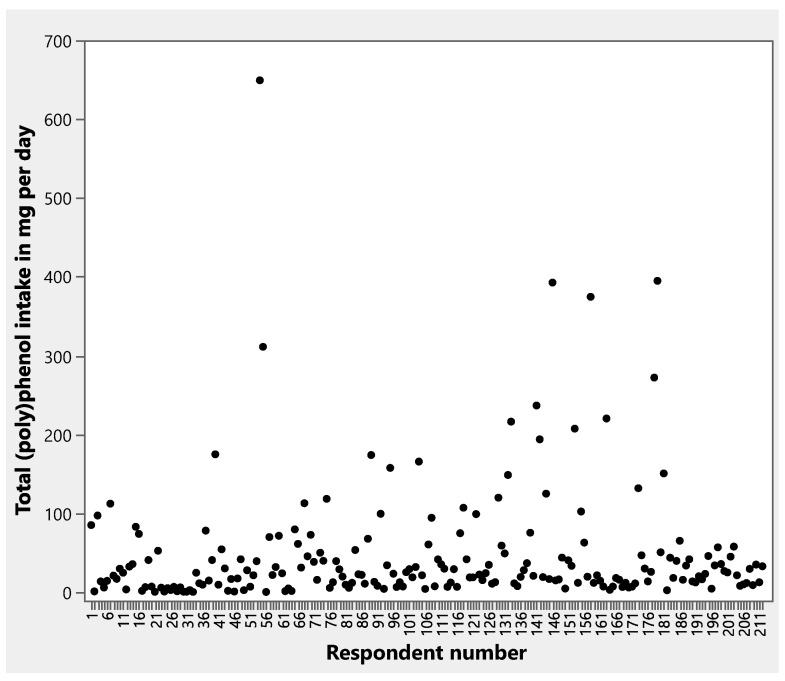
Scatter plot of total (poly)phenol intakes from herbs and spices per day per respondent.

Total (poly)phenol intakes from all herbs and spices and from individual herbs and spices are shown in Table 5 and Table 6. Cloves and cinnamon contributed the greatest amounts of total (poly)phenols to respondents’ intakes (Table 6), reflecting the relatively high concentrations of (poly)phenols in these spices (Figure 1). A scatter plot depicts the distribution of total daily (poly)phenol intakes for respondents (Figure 2). (Poly)phenol subclass intakes are shown in Supplementary Table S1. Of the flavonoids, flavanones were consumed in greater amounts than flavones or flavonols. Phenolic acids, phenolic terpenes, and lignans contributed smaller levels of (poly)phenols to intakes compared to flavonoids. The highest intakes were seen within the Phenol-Explorer database group of other polyphenols, which were primarily the result of high levels of hydroxyphenylpropenes in cloves and curcuminoids in turmeric.

The contribution of each herb and spice to total (poly)phenol intake is presented in Table 7. Black pepper and cinnamon, which were also among the most commonly consumed herbs and spices, contributed the most to total (poly)phenol intake from herbs and spices.

## 4. Discussion

This is the first known report of total (poly)phenol intakes calculated from herb and spice intakes collected from respondents completing a questionnaire. These findings show that culinary herbs and spices contribute a potentially meaningful level of total (poly)phenols to dietary intakes. The estimated total (poly)phenol daily intakes from herbs and spices presented here represent 3–12% of those reported from food and beverages in large-cohort studies. For example, Huang et al. investigated the US National Health and Nutrition Examination Survey (NHANES) 2013–2016 cohort, which employed two 24 h dietary recalls and showed a mean total (poly)phenol intake of 1656 (SE 35) mg/d [13]. Burkholder-Cooley et al. found a mean total (poly)phenol intake of 801 (SD 356) mg/d from the Adventist Health Study-2 that utilized a food-frequency questionnaire (FFQ) [14]. Pérez-Jiménez et al. studied a French cohort within the SUpplémentation en VItamines et Minéraux AntioXydants study (SU.VI.MAX), which used six 24 h dietary records to show a mean total (poly)phenol intake of 1193 (SD 510) mg/d [15]. Using 24 h recalls, the Canadian Community Health Survey 2015 cohort of adults was shown to consume 1119 mg/1000 kcal/d total (poly)phenols [16]. Zamora-Ros et al. analyzed FFQ data from the Mexican Teachers’ Cohort and reported total (poly)phenol intakes of 750 mg/d in Baja California, 746 mg/d in Mexico City, and 536 mg/d in Yucatan [17]. Miranda et al. reported an average total (poly)phenol intake of 377 mg/d in Brazilian adults who completed one 24 h recall [18]. These studies used Phenol-Explorer to determine (poly)phenol intakes from foods and beverages.

The influence of nationality on diet and (poly)phenol intake is evident in the aforementioned cohort studies and a systematic review by Del Bo et al. [5], who reported intakes of (poly)phenols and their subclasses, as well as food and beverage sources, from 45 publications. Most of the studies reviewed were performed in North America, Asia, and Europe. In the USA, total (poly)phenol intakes were shown to average 402–1370 mg/d and flavonoid intakes 189–209 mg/d, with the main sources being tea, coffee, wine, fruit, vegetables, and legumes (herbs/spices not measured) [14,19,20,21,22]. In Poland, total flavonoid intakes were reported as 610–622 mg (median) and total (poly)phenols as averaging 989–1740 mg, with the top sources being tea, fruit, vegetables, chocolate, and cereal [8,23,24,25]. In Japan, total flavanols were reported to average 1277 mg and total (poly)phenols 1492 mg, with the main contributors being green tea, onion, soy products, and coffee [26,27]. In Korea, total flavonoids were shown to have a mean of 96.6 mg with the main sources being kimchi, green tea, soy, onion, and fruit [28]. Populations in China were reported to consume total flavonoids at 20–50 mg/d and total flavanols at 19 mg/d, mostly from vegetables, fruit, and soybean sprouts [29,30,31]. Across multiple European countries, mean total (poly)phenol intake was estimated at 329 mg/d, with main sources being fruit, juices, and chocolate [32]. Notably, none of these studies reported assessing herb and spice intake.

Herbs and spices are concentrated sources of (poly)phenols, and if these cohort studies had collected data on herb and spice consumption, total dietary intakes of (poly)phenols likely would have been higher than those reported for foods and beverages alone [9]. The magnitude of increase in (poly)phenol intakes resulting from the inclusion of herbs and spices would depend on population, since herb/spice use varies across countries and regions, with the Caribbean, South Asian, and African nations showing the highest per capita consumption rates [33,34,35]. The use of culinary herb and spice mixtures characterizes a people group’s culture, often being linked with rituals and ceremonies. Examples include Indian masalas, Chinese Five-Spice, Middle Eastern za’atar, Mexican mole, Caribbean Jerk spice blend, Ethiopian Bebere, Japanese Shichimi, Italian seasoning, and French Herbes de Provence [36]. Groups that habitually consume herb/spice blends would be expected to have relatively high intakes of (poly)phenols.

Several publications have reported herb and spice intakes that were similar or moderately higher that those reported in this study. One publication is from Carlsen et al., who examined herb and spice consumption in 146 Norwegian adults and found daily intakes of 2.7 g/d (4.4) median (IQR) from an FFQ and 1.6 g/d (1.8) from a herb/spice record [37]. These findings, specifically from Carlsen et al.’s herb/spice record [(1.6 g/d (1.8)], are nearly identical to the those from the current study [(1.58 g/d (2.02)]. Sasaki et al. conducted an FFQ validation study in 215 men and women enrolled in the Japan Public Health Center-based Prospective Study and reported mean seasoning/spice intakes of 5 ± 5 g/d from an FFQ [38]. In another FFQ validation study, Pelligrini et al. surveyed 285 Italian men and women and showed spice intakes as 3.2 (2.7) g/d median (IQR) and 0.4 (1.3) g/d from the FFQ and 3-day weighed food records, respectively [39].

Two investigations from India reported substantially larger spice intakes compared to the current study. Uma Pradeep et al. interviewed 100 housewives in India for household spice intake and showed daily intakes of 9.54 ± 10.11 g spices per day per “consumption unit” or coefficient of an adult Indian man [40]. Uma Pradeep et al.’s findings likely reflect the higher spice use in India versus the United States [34]. When examining intakes of individual spices common to Uma Pradeep et al. and the current study, similar consumption levels are seen for black pepper at 0.33 ± 0.30 g/d and 0.35 ± 0.58 g/d, respectively, but lower intakes are seen for dried ginger (0.04 ± 1.31 g/d and 0.31 ± 0.60 g/d, respectively). Lastly, Bhathal et al. measured spice intakes in 100 urban and 100 rural households in Punjab, India, and reported mean spice intakes of 10.04 g/d and 7.68 g/d for urban and rural women, respectively [41]. Supplementary Table S2 shows a comparison of herb and/or spice intakes across studies.

The current study focused upon total (poly)phenols consumed in herbs and spices as total (poly)phenols, but not (poly)phenol classes and subclasses, are consistently reported in Phenol-Explorer. (Poly)phenol classes and subclasses including phenolic acids (hydroxybenzoic and hydroxycinnamic acids), flavonoids (flavonols, flavones, isoflavonoids, flavanones, and anthocyanidins), lignans, and stilbenes have been identified in select herbs and spices, but comprehensive quantitative data for herbs and spices are not currently available [42,43,44,45,46,47]. Therefore, the (poly)phenol subclass intakes reported in this study are likely underestimates.

Herbs and spices contribute nutrients beyond (poly)phenols to the diet, albeit generally in small amounts [48,49]. The concentration of secondary metabolites in plants is affected by environmental conditions such as light, temperature, ozone, carbon dioxide, and soil pH and salinity [50], but databases including the USDA FoodData Central [51] provide estimates of the nutrient composition of herbs and spices. Select micronutrients found in herbs and spices include calcium (22 mg/g basil), iron (0.5 mg/g turmeric), magnesium (7 mg/g basil), potassium (11 mg/g curry), vitamin A (0.15 µg RAE/g cinnamon), vitamin E (0.25 mg/g curry), folate (3 µg/g rosemary) and vitamin K (phylloquinone, 17.1 µg/g thyme). Including herb and spice assessment in dietary studies would allow for the identification of not only (poly)phenols but also essential micronutrients contributed by herbs and spices.

In relation to intake of herbs and spices assessed by our herb and spice questionnaire, the current results replicate some of those from our prior study [11]. Both studies identified the most commonly consumed herbs and spices as black pepper, garlic, and cinnamon at similar frequencies. Both studies also reported the median and mean number of herbs and spices consumed per month as ~8. However, the respondents in the current compared to the previous study reported higher levels of herb and spice consumption [current study g/mo, median (IQR): 47.44 (60.71) versus prior study: 26.9 (41.4) and 36.5 (42.9)]. The larger sample size in the current versus previous study (*n* = 212 vs. 62) captured a greater representation of intakes and may account, at least in part, for the higher intake values.

This study utilized a frequency questionnaire as the assessment tool. Because herbs and spices are typically consumed in relatively small amounts and sporadically, the herb and spice questionnaire queried frequency and amounts of intakes over the past 30 days. Multiple methods of dietary assessment exist, each with its strengths and limitations [52]. The 24 h dietary recalls commonly used for nutrition assessment in cohort studies would likely not be appropriate for evaluating habitual herb and spice intake. Food-frequency questionnaires assess dietary intakes during the past 6 or 12 months, and this method could readily incorporate questions about herb and spice intake. A combination of 24 h recalls or diet records and a herb/spice frequency questionnaire is also an option for including herb and spice assessment in cohort nutrition studies.

The herb and spice questionnaire used in this study included fresh and dried options of most items, and this is important when assessing (poly)phenol concentrations. Substantial differences exist in (poly)phenol content and antioxidant activity between fresh and dried herbs and spices [9,53]. Another aspect of the (poly)phenol content and antioxidant potency of herb and spice intake to consider is preparation/cooking method and cooking time. Heat treatment increases the phenol content and radical scavenging activity of many herbs and spices [54]. Moisture, heat, and time factors may also affect the (poly)phenol content of foods prepared with herbs and spices, such as vegetables [55,56,57]. A future version of the herb and spice questionnaire could include probes about how herbs and spices were used during food preparation and cooking. To be of value, however, this information about herb/spice exposure to cooking would need to be combined with experimental data on the heat-induced changes to (poly)phenol content.

A recent advancement of dietary assessment is biomarkers of intake/exposure, which seek to objectively measure dietary intakes [58,59]. Biomarkers of herb and spice intake have been characterized, but most of the investigations producing these data used purified, encapsulated/tablet forms of the herb/spice that may not represent the absorption and metabolism of herbs and spices in whole-food diets [60]. Identifying biomarkers of habitual herb and spice intake and the (poly)phenol metabolites could advance efforts to determine the association of diet and health outcomes.

An important aspect affecting the biomarkers and physiologic effects of dietary (poly)phenols is their absorption, metabolism, pharmacodynamics, and pharmacokinetics [61,62,63]. The absorption of dietary (poly)phenols is affected by the matrix of the source, other compounds in the meal such as fiber and fat, bound molecular groups such as sugars and esters, and uptake, metabolism, and secretion into the lumen by enterocytes [64,65]. Phenolic compounds not absorbed in the upper gastrointestinal tract or secreted in bile undergo metabolism by the colonic microbiota and these metabolites can be absorbed by the host. Phenolic compounds absorbed by enterocytes are conjugated with glucuronide, methyl, and sulfate molecules by the intestine and liver. Metabolites of phenolic compounds circulate in blood and can be taken up by tissues to exert biological effects [66,67]. Notably, humans display inter-individual variation in (poly)phenol absorption and pharmacokinetic fate, which may be a function of differences in microbiota profile, sex, and/or polymorphisms in genes coding for transporters or enzymes of metabolism [68,69]. The nature of (poly)phenol absorption, metabolism, and distribution presents multiple complexities in understanding the path from herb/spice consumption to health outcomes.

Strengths of this study include the detailed assessment of herb and spice intake in a sample of >200 adults and the use of Phenol-Explorer, an established database of food (poly)phenol concentrations. Limitations include the absence of information on (poly)phenol intakes from food and beverages in the respondents. The decision not to collect full dietary data was based on concerns of creating a participant burden that would discourage participation in the main focus of the study, assessment of herb and spice consumption. Another limitation is the absence of complete data on specific (poly)phenol subclass intake [53]. Phenol-Explorer consistently reports total (poly)phenol measurements, but not comprehensive (poly)phenol class and subclass values, for herbs and spices [70]. The use of the Folin–Ciocalteu method for analyzing total (poly)phenols in the Phenol-Explorer database is an additional limitation. This method is shown to be non-specific to (poly)phenols and may overestimate phenolic measurements due to interference from other reducing compounds such as ascorbic acid [71,72,73]. Gas chromatography or high-performance liquid chromatography coupled with mass spectrometry are more reliable, specific, and sensitive for characterizing phenolic compounds [74].

## 5. Conclusions

This study of 212 adults showed monthly intakes of 679.92 (1134.06) (median, IQR) mg total (poly)phenols from 47.44 (60.71) g herbs and spices. Considering the evidence supporting the beneficial effect of (poly)phenols on multiple health outcomes, investigators should consider incorporating herb and spice assessment in cohort nutrition and health studies.

## Figures and Tables

**Table 1:** Herbs and spices included in the questionnaire.

Anise Herb, Dried *Pimpinella Anisum*	Basil, Sweet, Dried *Ocimum Basilicum*	Basil, Sweet, Fresh *Ocimum Basilicum*
Black pepper, Pepper *Piper nigrum*	Cinnamon *Cinnamomum* spp.	Clove *Syzygium aromaticum*
Common sage, dried *Salvia ocinalis*	Common sage, fresh *Salvia ocinalis*	Common thyme, dried *Thymus vulgaris*
Common thyme, fresh *Thymus vulgaris*	Cumin *Cuminum cyminum*	Curry, powder *Murraya koenigii*
Garlic, fresh *Allium sativum*	Ginger, dried *Zingiber officinale*	Ginger, fresh *Zingiber officinale*
Marjoram, dried *Origanum majorana*	Marjoram, fresh *Origanum majorana*	Nutmeg *Myristica fragrans*
Oregano, dried *Origanum* spp.	Oregano, fresh *Origanum* spp.	Parsley, dried *Petroselinum crispum*
Parsley, fresh *Petroselinum crispum*	Peppermint, fresh *Mentha* spp.	Rosemary, dried *Rosmarinus officinalis*
Rosemary, fresh *Rosmarinus officinalis*	Spearmint, fresh *Mentha* spp.	Turmeric, dried *Curcuma longa*

spp.: species.

**Table 2:** Herb and spice total intake per respondent, g.

	Median (IQR)	Mean (SD)
Per month	47.44 (60.71)	72.19 (86.59)
Per day	1.58 (2.02)	2.41 (2.89)

Total number of respondents: 212. IQR: interquartile range; SD: standard deviation.

**Table 3:** Frequencies of consumption of specific herbs and spices.

Herb/Spice	*n* Reporting Intake	% Reporting Intake
Anise, dried	19	9.00%
Basil, sweet, dried	89	42.00%
Basil, sweet, fresh	84	39.60%
Black pepper	170	80.20%
Cinnamon	136	64.20%
Cloves	50	23.60%
Cumin	115	54.20%
Curry	60	28.30%
Garlic, fresh	143	67.50%
Ginger, dried	64	30.20%
Ginger, fresh	73	34.40%
Marjoram, dried	13	6.10%
Marjoram, fresh	10	4.70%
Mint, peppermint	41	19.30%
Mint, spearmint	26	12.30%
Nutmeg	55	25.90%
Oregano, dried	121	57.10%
Oregano, fresh	28	13.20%
Parsley, dried	82	38.70%
Parsley, fresh	57	26.90%
Rosemary, dried	77	36.30%
Rosemary, fresh	31	14.60%
Sage, dried	32	15.10%
Sage, fresh	13	6.10%
Thyme, dried	87	41.00%
Thyme, fresh	20	9.40%
Turmeric, dried	72	34.00%

Total number of respondents: 212.

**Table 4:** Amount of herb/spice consumed, g/month and g/day.

	g Per Month		g Per Day	
Herb/Spice	Median (IQR)	Mean (SD)	Median (IQR)	Mean (SD)
Anise, dried	1.15 (13.23)	10.47 (23.36)	0.44 (0.66)	0.87 (1.49)
Basil, sweet, dried	1.26 (2.30)	2.58 (3.26)	0.32 (0.54)	0.53 (0.58)
Basil, sweet, fresh	2.28 (4.42)	3.85 (4.46)	0.15 (0.37)	0.33 (0.48)
Black pepper	13.11 (19.80)	26.08 (44.72)	0.13 (0.34)	0.35 (0.58)
Cinnamon	2.69 (6.00)	7.80 (14.65)	0.10 (0.15)	0.31 (0.70)
Cloves	2.06 (4.43)	6.00 (15.30)	0.10 (0.53)	0.37 (0.51)
Cumin	3.05 (4.57)	9.35 (21.04)	0.10 (0.29)	0.21 (0.25)
Curry	2.53 (6.61)	14.87 (58.89)	0.09 (0.20)	0.26 (0.49)
Garlic, fresh	9.52 (16.32)	15.93 (17.25)	0.08 (0.22)	0.5 (1.96)
Ginger, dried	1.43 (3.57)	5.34 (10.03)	0.08 (0.22)	0.31 (0.60)
Ginger, fresh	4.40 (11.00)	10.05 (14.54)	0.08 (0.15)	0.13 (0.15)
Marjoram, dried	0.17 (0.38)	0.37 (0.50)	0.07 (0.15)	0.20 (0.51)
Marjoram, fresh	1.72 (3.41)	2.26 (2.48)	0.06 (0.11)	0.08 (0.08)
Mint, peppermint	0.80 (3.08)	3.08 (6.04)	0.05 (0.19)	0.16 (0.35)
Mint, spearmint	1.59 (5.83)	4.90 (10.40)	0.05 (0.12)	0.18 (0.33)
Nutmeg	1.05 (2.18)	3.03 (6.49)	0.05 (0.07)	0.09 (0.14)
Oregano, dried	1.35 (2.02)	2.70 (4.09)	0.04 (0.08)	0.09 (0.11)
Oregano, fresh	3.90 (10.23)	10.65 (17.30)	0.04 (0.44)	0.35 (0.78)
Parsley, dried	0.49 (1.40)	0.94 (1.54)	0.03 (0.07)	0.10 (0.22)
Parsley, fresh	2.38 (6.64)	9.26 (17.96)	0.03 (0.08)	0.14 (0.45)
Rosemary, dried	0.64 (3.59)	3.59 (7.44)	0.03 (0.10)	0.10 (0.20)
Rosemary, fresh	0.72 (1.80)	2.50 (4.27)	0.02 (0.06)	0.08 (0.14)
Sage, dried	0.35 (0.55)	2.30 (5.51)	0.02 (0.12)	0.12 (0.25)
Sage, fresh	0.52 (2.83)	2.79 (5.16)	0.02 (0.09)	0.09 (0.17)
Thyme, dried	0.98 (2.46)	4.24 (13.36)	0.02 (0.05)	0.03 (0.05)
Thyme, fresh	2.93 (8.77)	6.25 (7.49)	0.01 (0.02)	0.08 (0.18)
Turmeric, dried	3.03 (15.98)	11.16 (15.18)	0.01 (0.01)	0.01 (0.02)

**Table 5:** Total (poly)phenol intake from herbs and spices, mg.

	Median (IQR)	Mean (SD)
Per month	679.92 (1134.06)	1143.31 (2327.41)
Per day	22.66 (37.81)	48.11 (77.58)

**Table 6:** Total (poly)phenols consumed from specific herbs and spices.

	mg Per Month		mg Per Day	
Herb/Spice	Median (IQR)	Mean (SD)	Median (IQR)	Mean (SD)
Anise, dried	2.07 (23.81)	18.84 (42.05)	11.04 (23.71)	32.09 (81.83)
Basil, sweet, dried	54.58 (99.08)	111.52 (140.55)	8.71 (19.40)	25.21 (47.37)
Basil, sweet, fresh	5.28 (10.23)	8.92 (10.33)	4.37 (6.60)	8.69 (14.91)
Black pepper	131.1 (198.00)	260.78 (447.23)	3.48 (12.78)	10.74 (22.80)
Cinnamon	261.32 (582.05)	756.32 (1421.21)	2.14 (11.28)	7.87 (10.71)
Cloves	331.21 (711.33)	962.79 (2454.84)	2.07 (3.11)	6.35 (14.29)
Cumin	62.24 (93.18)	190.59 (428.73)	1.82 (3.30)	3.72 (4.69)
Curry	27.24 (71.01)	159.88 (633.05)	1.40 (2.10)	2.81 (4.25)
Garlic, fresh	8.29 (14.21)	13.86 (15.02)	1.22 (3.19)	3.32 (5.39)
Ginger, dried	6.75 (16.86)	25.25 (47.46)	1.14 (3.43)	2.44 (2.93)
Ginger, fresh	8.98 (22.44)	20.50 (29.67)	0.91 (2.37)	5.33 (21.10)
Marjoram, dried	6.62 (14.57)	14.36 (19.13)	0.67 (1.38)	1.92 (4.12)
Marjoram, fresh	14.71 (29.10)	19.31 (21.19)	0.59 (1.49)	2.56 (8.08)
Mint, peppermint	7.79 (30.14)	30.15 (59.17)	0.54 (3.01)	3.01 (6.24)
Mint, spearmint	104.54 (383.32)	322.21 (683.98)	0.49 (0.97)	0.64 (0.71)
Nutmeg	19.96 (41.52)	57.74 (123.64)	0.34 (0.54)	2.24 (5.36)
Oregano, dried	42.08 (62.93)	84.25 (127.49)	0.30 (0.75)	0.68 (0.99)
Oregano, fresh	36.47 (95.61)	99.53 (161.71)	0.28 (0.47)	0.46 (0.50)
Parsley, dried	7.68 (22.19)	14.84 (24.43)	0.26 (0.65)	0.90 (1.54)
Parsley, fresh	2.12 (5.91)	8.24 (15.98)	0.26 (1.00)	1.00 (1.97)
Rosemary, dried	16.12 (90.35)	90.40 (187.28)	0.26 (0.74)	0.49 (0.81)
Rosemary, fresh	7.80 (19.50)	27.08 (46.21)	0.22 (0.56)	0.84 (1.58)
Sage, dried	10.22 (16.07)	67.11 (160.82)	0.22 (0.49)	0.48 (0.64)
Sage, fresh	0.95 (5.25)	5.16 (9.55)	0.18 (0.34)	0.30 (0.34)
Thyme, dried	17.71 (44.65)	76.88 (242.44)	0.07 (0.20)	0.27 (0.53)
Thyme, fresh	34.35 (102.90)	73.28 (87.80)	0.07 (0.79)	0.63 (1.40)
Turmeric, dried	64.18 (338.31)	236.17 (321.3)	0.03 (0.17)	0.17 (0.32)

**Table 7:** Contribution of specific herbs and spices to total (poly)phenol intake from herbs and spices.

Herb/Spice	Median (IQR)	Mean (SD)
Anise, dried	0.60 (3.00)%	18.59 (38.48)%
Basil, sweet, dried	5.80 (11.95)%	12.12 (16.60)%
Basil, sweet, fresh	0.70 (1.25)%	2.42 (11.06)%
Black pepper	23.90 (35.40)%	30.71 (27.19)%
Cinnamon	31.75 (40.03)%	34.77 (25.02)%
Cloves	14.30 (30.60)%	27.02 (28.84)%
Cumin	7.30 (12.90)%	14.33 (19.80)%
Curry	4.50 (6.55)%	6.94 (7.33)%
Garlic, fresh	0.90 (1.90)%	2.28 (4.19)%
Ginger, dried	1.05 (2.73)%	3.78 (9.73)%
Ginger, fresh	0.80 (2.08)%	3.73 (12.34)%
Marjoram, dried	0.50 (2.68)%	1.35 (1.39)%
Marjoram, fresh	1.45 (53.85)%	25.18 (41.19)%
Mint, peppermint	1.50 (2.50)%	6.05 (15.37)%
Mint, spearmint	8.25 (16.05)%	17.84 (26.76)%
Nutmeg	1.90 (3.45)%	4.59 (9.02)%
Oregano, dried	5.30 (7.75)%	8.57 (11.10)%
Oregano, fresh	2.65 (12.53)%	7.78 (10.91)%
Parsley, dried	0.85 (1.93)%	2.35 (4.43)%
Parsley, fresh	0.65 (0.85)%	1.01 (1.21)%
Rosemary, dried	1.90 (7.70)%	6.97 (11.18)%
Rosemary, fresh	0.90 (1.90)%	2.14 (3.06)%
Sage, dried	0.90 (1.90)%	4.44 (9.41)%
Sage, fresh	0.30 (1.60)%	1.86 (3.94)%
Thyme, dried	2.00 (4.90)%	5.74 (12.43)%
Thyme, fresh	4.00 (8.10)%	6.82 (8.09)%
Turmeric, dried	6.20 (22.80)%	19.72 (27.30)%

Total (poly)phenol intakes (mg/mo) from individual herbs and spices were significantly different, Chi-square *p* < 0.0001. Nonparametric rank sum pairwise comparisons showed no significant differences in (poly)phenol intakes between cinnamon and cloves and between cumin and turmeric, *p* > 0.05.

## Data Availability

The data supporting the conclusions of this article will be made available by the author on reasonable request due to privacy reasons.

## Supplementary Materials

The following supporting information can be downloaded at https://www.mdpi.com/article/10.3390/nu17152445/s1, Table S1: (Poly)phenol subclass intakes from herbs and spices, mg per month, *n* = 212 respondents; Table S2: Comparison of herb and spice intakes across studies.

## References

[1] Tapsell L.C., Hemphill I., Cobiac L., Patch C.S., Sullivan D.R., Fenech M., Roodenrys S., Keogh J.B., Clifton P.M., Williams P.G. (2006). Health Benefits of Herbs and Spices: The Past, the Present, the Future. Med. J. Aust..

[2] Spence C. (2024). Unveiling the Health-Promoting Power of Bioactive Compounds in Herbs and Spices. Curr. Food Sci. Technol. Rep..

[3] Jiang T.A. (2019). Health Benefits of Culinary Herbs and Spices. J. AOAC Int..

[4] Al-Habsi N., Al-Khalili M., Haque S.A., Al Akhzami N., Gonzalez-Gonzalez C.R., Al Harthi S., Al Jufaili S.M. (2025). Herbs and Spices as Functional Food Ingredients: A Comprehensive Review of Their Therapeutic Properties, Antioxidant and Antimicrobial Activities, and Applications in Food Preservation. J. Funct. Foods.

[5] Del Bo’ C., Bernardi S., Marino M., Porrini M., Tucci M., Guglielmetti S., Cherubini A., Carrieri B., Kirkup B., Kroon P. (2019). Systematic Review on Polyphenol Intake and Health Outcomes: Is There Sufficient Evidence to Define a Health-Promoting Polyphenol-Rich Dietary Pattern?. Nutrients.

[6] Zamora-Ros R., Forouhi N.G., Sharp S.J., González C.A., Buijsse B., Guevara M., van der Schouw Y.T., Amiano P., Boeing H., Bredsdorff L. (2013). The Association between Dietary Flavonoid and Lignan Intakes and Incident Type 2 Diabetes in European Populations: The EPIC-InterAct Study. Diabetes Care.

[7] Tresserra-Rimbau A., Rimm E.B., Medina-Remón A., Martínez-González M.A., de la Torre R., Corella D., Salas-Salvadó J., Gómez-Gracia E., Lapetra J., Arós F. (2014). Inverse Association between Habitual Polyphenol Intake and Incidence of Cardiovascular Events in the PREDIMED Study. Nutr. Metab. Cardiovasc. Dis..

[8] Grosso G., Stepaniak U., Micek A., Stefler D., Bobak M., Pająk A. (2017). Dietary Polyphenols Are Inversely Associated with Metabolic Syndrome in Polish Adults of the HAPIEE Study. Eur. J. Nutr..

[9] Bieżanowska-Kopeć R., Piątkowska E. (2022). Total Polyphenols and Antioxidant Properties of Selected Fresh and Dried Herbs and Spices. Appl. Sci..

[10] (2024). Fact.MR Spice and Herb Market Study by Spices and Herbs for Food, Beverages, Foodservice, Retail/Household, and Personal Care and Cosmetics from 2024 to 2034. https://www.factmr.com/report/spice-and-herb-market.

[11] Blanton C. (2020). Relative Validity of an Online Herb and Spice Consumption Questionnaire. Int. J. Environ. Res. Public Health.

[12] Neveu V., Perez-Jiménez J., Vos F., Crespy V., du Chaffaut L., Mennen L., Knox C., Eisner R., Cruz J., Wishart D. (2010). Phenol-Explorer: An Online Comprehensive Database on Polyphenol Contents in Foods. Database J. Biol. Databases Curation.

[13] Huang Q., Braffett B.H., Simmens S.J., Young H.A., Ogden C.L. (2020). Dietary Polyphenol Intake in US Adults and 10-Year Trends: 2007–2016. J. Acad. Nutr. Diet..

[14] Burkholder-Cooley N., Rajaram S., Haddad E., Fraser G.E., Jaceldo-Siegl K. (2016). Comparison of Polyphenol Intakes According to Distinct Dietary Patterns and Food Sources in the Adventist Health Study-2 Cohort. Br. J. Nutr..

[15] Pérez-Jiménez J., Fezeu L., Touvier M., Arnault N., Manach C., Hercberg S., Galan P., Scalbert A. (2011). Dietary Intake of 337 Polyphenols in French Adults. Am. J. Clin. Nutr..

[16] Biancaniello E.C., Tiessen S., Hartman B., Battram D.S. (2024). Dietary Polyphenol Intake in the Canadian Population: Findings from the 2015 Canadian Community Health Survey-Nutrition. Can. J. Public Health Rev. Can. Sante Publique.

[17] Zamora-Ros R., Biessy C., Rothwell J.A., Monge A., Lajous M., Scalbert A., López-Ridaura R., Romieu I. (2018). Dietary Polyphenol Intake and Their Major Food Sources in the Mexican Teachers’ Cohort. Br. J. Nutr..

[18] Miranda A.M., Steluti J., Fisberg R.M., Marchioni D.M. (2016). Dietary Intake and Food Contributors of Polyphenols in Adults and Elderly Adults of Sao Paulo: A Population-Based Study. Br. J. Nutr..

[19] Song W.O., Chun O.K. (2008). Tea Is the Major Source of Flavan-3-Ol and Flavonol in the U.S. Diet. J. Nutr..

[20] Chun O.K., Floegel A., Chung S.-J., Chung C.E., Song W.O., Koo S.I. (2010). Estimation of Antioxidant Intakes from Diet and Supplements in U.S. Adults. J. Nutr..

[21] Sebastian R.S., Wilkinson Enns C., Goldman J.D., Martin C.L., Steinfeldt L.C., Murayi T., Moshfegh A.J. (2015). A New Database Facilitates Characterization of Flavonoid Intake, Sources, and Positive Associations with Diet Quality among US Adults. J. Nutr..

[22] Kim K., Vance T.M., Chun O.K. (2016). Estimated Intake and Major Food Sources of Flavonoids among US Adults: Changes between 1999–2002 and 2007–2010 in NHANES. Eur. J. Nutr..

[23] Ilow R., Regulska-Ilow B., Rózańska D., Misiewicz D., Grajeta H., Kowalisko A., Biernat J. (2012). Assessment of Dietary Flavonoid Intake among 50-Year-Old Inhabitants of Wroclaw in 2008. Adv. Clin. Exp. Med. Off. Organ Wroclaw Med. Univ..

[24] Zujko M.E., Witkowska A.M., Waśkiewicz A., Sygnowska E. (2012). Estimation of Dietary Intake and Patterns of Polyphenol Consumption in Polish Adult Population. Adv. Med. Sci..

[25] Witkowska A.M., Zujko M.E., Waśkiewicz A., Terlikowska K.M., Piotrowski W. (2015). Comparison of Various Databases for Estimation of Dietary Polyphenol Intake in the Population of Polish Adults. Nutrients.

[26] Otaki N., Kimira M., Katsumata S.-I., Uehara M., Watanabe S., Suzuki K. (2009). Distribution and Major Sources of Flavonoid Intakes in the Middle-Aged Japanese Women. J. Clin. Biochem. Nutr..

[27] Taguchi C., Fukushima Y., Kishimoto Y., Suzuki-Sugihara N., Saita E., Takahashi Y., Kondo K. (2015). Estimated Dietary Polyphenol Intake and Major Food and Beverage Sources among Elderly Japanese. Nutrients.

[28] Kim Y.J., Park M.Y., Chang N., Kwon O. (2015). Intake and Major Sources of Dietary Flavonoid in Korean Adults: Korean National Health and Nutrition Examination Survey 2010–2012. Asia Pac. J. Clin. Nutr..

[29] Zhang Y., Cao J., Chen W., Yang J., Hao D., Zhang Y., Chang P., Zhao X. (2010). Reproducibility and Relative Validity of a Food Frequency Questionnaire to Assess Intake of Dietary Flavonol and Flavone in Chinese University Campus Population. Nutr. Res..

[30] Zhang Y., Li Y., Cao C., Cao J., Chen W., Zhang Y., Wang C., Wang J., Zhang X., Zhao X. (2010). Dietary Flavonol and Flavone Intakes and Their Major Food Sources in Chinese Adults. Nutr. Cancer.

[31] Sun C., Wang H., Wang D., Chen Y., Zhao Y., Xia W. (2015). Using an FFQ to Assess Intakes of Dietary Flavonols and Flavones among Female Adolescents in the Suihua Area of Northern China. Public Health Nutr..

[32] Wisnuwardani R.W., De Henauw S., Androutsos O., Forsner M., Gottrand F., Huybrechts I., Knaze V., Kersting M., Le Donne C., Marcos A. (2019). Estimated Dietary Intake of Polyphenols in European Adolescents: The HELENA Study. Eur. J. Nutr..

[33] (2025). Spices and Seasonings Market Size, Share & Industry Analysis, By Type (Pepper, Chili, Ginger, Cinnamon, Cumin, Turmeric, Nutmeg and Mace, Cardamom, Cloves, and Others), By Application (Meat and Poultry, Bakery and Confectionery, Frozen Food, Snacks & Convenience Food, and Others), and Regional Forecast, 2024–2032. https://www.fortunebusinessinsights.com/industry-reports/spices-and-seasonings-market-101694.

[34] (2025). Hegli Library Spice Consumption per Capita. https://www.helgilibrary.com/indicators/spice-consumption-per-capita/.

[35] Grivetti L. (2016). Herbs, Spices, and Flavoring Agents: Part 1: Old World Contributions. Nutr. Today.

[36] Mathea Ford Herbs & Spices, Spices and Culture: 11 Diverse Culinary Practices 2024. https://usingherbsandspices.com/spices-and-culture-11-diverse-culinary-practices/.

[37] Carlsen M.H., Blomhoff R., Andersen L.F. (2011). Intakes of Culinary Herbs and Spices from a Food Frequency Questionnaire Evaluated against 28-Days Estimated Records. Nutr. J..

[38] Sasaki S., Kobayashi M., Tsugane S. (2003). Validity of a Self-Administered Food Frequency Questionnaire Used in the 5-Year Follow-up Survey of the JPHC Study Cohort I: Comparison with Dietary Records for Food Groups. J. Epidemiol..

[39] Pellegrini N., Salvatore S., Valtueña S., Bedogni G., Porrini M., Pala V., Del Rio D., Sieri S., Miglio C., Krogh V. (2007). Development and Validation of a Food Frequency Questionnaire for the Assessment of Dietary Total Antioxidant Capacity. J. Nutr..

[40] Uma Pradeep K., Geervani P., Eggum B.O. (1993). Common Indian Spices: Nutrient Composition, Consumption and Contribution to Dietary Value. Plant Foods Hum. Nutr. Dordr. Neth..

[41] Bhathal S.K., Kaur H., Bains K., Mahal A.K. (2020). Assessing Intake and Consumption Level of Spices among Urban and Rural Households of Ludhiana District of Punjab, India. Nutr. J..

[42] Chociej P., Foss K., Jabłońska M., Ustarbowska M., Sawicki T. (2024). The Profile and Content of Polyphenolic Compounds and Antioxidant and Anti-Glycation Properties of Root Extracts of Selected Medicinal Herbs. Plant Foods Hum. Nutr. Dordr. Neth..

[43] Szydłowska-Czerniak A., Kowaluk A., Strzelec M., Sawicki T., Tańska M. (2025). Evaluation of Bioactive Compounds and Chemical Elements in Herbs: Effectiveness of Choline Chloride-Based Deep Eutectic Solvents in Ultrasound-Assisted Extraction. Molecules.

[44] Nakatani N. (2000). Phenolic Antioxidants from Herbs and Spices. BioFactors Oxf. Engl..

[45] Lin L.-Z., Harnly J.M. (2012). LC-PDA-ESI/MS Identification of the Phenolic Components of Three Compositae Spices: Chamomile, Tarragon, and Mexican Arnica. Nat. Prod. Commun..

[46] Cortés-Chitala M.D.C., Flores-Martínez H., Orozco-Ávila I., León-Campos C., Suárez-Jacobo Á., Estarrón-Espinosa M., López-Muraira I. (2021). Identification and Quantification of Phenolic Compounds from Mexican Oregano (Lippia Graveolens HBK) Hydroethanolic Extracts and Evaluation of Its Antioxidant Capacity. Molecules.

[47] Lazaridis D.G., Kitsios A.-P., Koutoulis A.S., Malisova O., Karabagias I.K. (2024). Fruits, Spices and Honey Phenolic Compounds: A Comprehensive Review on Their Origin, Methods of Extraction and Beneficial Health Properties. Antioxidants.

[48] Murphy E.W., Marsh A.C., Willis B.W. (1978). Nutrient Content of Spices and Herbs. J. Am. Diet. Assoc..

[49] Newerli-Guz J., Smiechowska M. (2024). Spices and Herbs Nomenclature—Current Results and Trends. Role and Importance in Developing the Quality of Spices and Herbs. Sci. Pap. Silesian Univ. Technol. Organ. Manag..

[50] Pant P., Pandey S., Dall’Acqua S. (2021). The Influence of Environmental Conditions on Secondary Metabolites in Medicinal Plants: A Literature Review. Chem. Biodivers..

[51] (2025). U.S. Department of Agriculture, Agricultural Research Service. FoodData Central. https://fdc.nal.usda.gov/.

[52] Bailey R.L. (2021). Overview of Dietary Assessment Methods for Measuring Intakes of Foods, Beverages, and Dietary Supplements in Research Studies. Curr. Opin. Biotechnol..

[53] Opara E.I., Chohan M. (2014). Culinary Herbs and Spices: Their Bioactive Properties, the Contribution of Polyphenols and the Challenges in Deducing Their True Health Benefits. Int. J. Mol. Sci..

[54] Khatun M., Eguchi S., Yamaguchi T., Takamura H., Matoba T. (2006). Effect of Thermal Treatment on Radical-Scavenging Activity of Some Spices. Food Sci. Technol. Res..

[55] Kim J.-S., Kang O.-J., Gweon O.-C. (2013). Comparison of Phenolic Acids and Flavonoids in Black Garlic at Different Thermal Processing Steps. J. Funct. Foods.

[56] Pellegrini N., Chiavaro E., Gardana C., Mazzeo T., Contino D., Gallo M., Riso P., Fogliano V., Porrini M. (2010). Effect of Different Cooking Methods on Color, Phytochemical Concentration, and Antioxidant Capacity of Raw and Frozen Brassica Vegetables. J. Agric. Food Chem..

[57] Palermo M., Pellegrini N., Fogliano V. (2014). The Effect of Cooking on the Phytochemical Content of Vegetables. J. Sci. Food Agric..

[58] Cuparencu C., Bulmuş-Tüccar T., Stanstrup J., La Barbera G., Roager H.M., Dragsted L.O. (2024). Towards Nutrition with Precision: Unlocking Biomarkers as Dietary Assessment Tools. Nat. Metab..

[59] Chakraborty H., Sun Q., Bhupathiraju S.N., Schenk J.M., Mishchuk D.O., Bain J.R., He X., Sun J., Harnly J., Simmons W. (2025). The Dietary Biomarkers Development Consortium: An Initiative for Discovery and Validation of Dietary Biomarkers for Precision Nutrition. Curr. Dev. Nutr..

[60] Vázquez-Fresno R., Rosana A.R.R., Sajed T., Onookome-Okome T., Wishart N.A., Wishart D.S. (2019). Herbs and Spices- Biomarkers of Intake Based on Human Intervention Studies—A Systematic Review. Genes Nutr..

[61] Stromsnes K., Lagzdina R., Olaso-Gonzalez G., Gimeno-Mallench L., Gambini J. (2021). Pharmacological Properties of Polyphenols: Bioavailability, Mechanisms of Action, and Biological Effects in In Vitro Studies, Animal Models, and Humans. Biomedicines.

[62] Matsui T. (2022). Polyphenols-Absorption and Occurrence in the Body System. Food Sci. Technol. Res..

[63] van Duynhoven J., Vaughan E.E., Jacobs D.M., Kemperman R.A., van Velzen E.J.J., Gross G., Roger L.C., Possemiers S., Smilde A.K., Doré J. (2011). Metabolic Fate of Polyphenols in the Human Superorganism. Proc. Natl. Acad. Sci. USA.

[64] Manach C., Scalbert A., Morand C., Rémésy C., Jiménez L. (2004). Polyphenols: Food Sources and Bioavailability. Am. J. Clin. Nutr..

[65] Zhang H., Yu D., Sun J., Liu X., Jiang L., Guo H., Ren F. (2014). Interaction of Plant Phenols with Food Macronutrients: Characterisation and Nutritional–Physiological Consequences. Nutr. Res. Rev..

[66] Maubach J., Bracke M.E., Heyerick A., Depypere H.T., Serreyn R.F., Mareel M.M., De Keukeleire D. (2003). Quantitation of Soy-Derived Phytoestrogens in Human Breast Tissue and Biological Fluids by High-Performance Liquid Chromatography. J. Chromatogr. B Analyt. Technol. Biomed. Life Sci..

[67] Alldritt I., Whitham-Agut B., Sipin M., Studholme J., Trentacoste A., Tripp J.A., Cappai M.G., Ditchfield P., Devièse T., Hedges R.E.M. (2019). Metabolomics Reveals Diet-Derived Plant Polyphenols Accumulate in Physiological Bone. Sci. Rep..

[68] Feliciano R.P., Mills C.E., Istas G., Heiss C., Rodriguez-Mateos A. (2017). Absorption, Metabolism and Excretion of Cranberry (Poly)Phenols in Humans: A Dose Response Study and Assessment of Inter-Individual Variability. Nutrients.

[69] Wruss J., Lanzerstorfer P., Huemer S., Himmelsbach M., Mangge H., Höglinger O., Weghuber D., Weghuber J. (2015). Differences in Pharmacokinetics of Apple Polyphenols after Standardized Oral Consumption of Unprocessed Apple Juice. Nutr. J..

[70] Singla R.K., Dubey A.K., Garg A., Sharma R.K., Fiorino M., Ameen S.M., Haddad M.A., Al-Hiary M. (2019). Natural Polyphenols: Chemical Classification, Definition of Classes, Subcategories, and Structures. J. AOAC Int..

[71] Khoddami A., Wilkes M.A., Roberts T.H. (2013). Techniques for Analysis of Plant Phenolic Compounds. Molecules.

[72] Raposo F., Borja R., Gutiérrez-González J.A. (2024). A Comprehensive and Critical Review of the Unstandardized Folin-Ciocalteu Assay to Determine the Total Content of Polyphenols: The Conundrum of the Experimental Factors and Method Validation. Talanta.

[73] Samara M., Nasser A., Mingelgrin U. (2022). Critical Examination of the Suitability of the Folin-Ciocalteu Reagent Assay for Quantitative Analysis of Polyphenols—The Case of Olive-Mill Wastewater. Am. J. Anal. Chem..

[74] Ali A., Wu H., Ponnampalam E.N., Cottrell J.J., Dunshea F.R., Suleria H.A.R. (2021). Comprehensive Profiling of Most Widely Used Spices for Their Phenolic Compounds through LC-ESI-QTOF-MS^2^ and Their Antioxidant Potential. Antioxidants.

